# Integrated Analysis of a Risk Score System Predicting Prognosis and a ceRNA Network for Differentially Expressed lncRNAs in Multiple Myeloma

**DOI:** 10.3389/fgene.2020.00934

**Published:** 2020-08-27

**Authors:** Sijie Zhou, Jiuyuan Fang, Yan Sun, Huixiang Li

**Affiliations:** ^1^The First Affiliated Hospital of Zhengzhou University, Zhengzhou, China; ^2^School of Basic Medical Sciences, Zhengzhou University, Zhengzhou, China

**Keywords:** long non-coding RNA, biomarkers, multiple myeloma, weighted gene co-expression network analysis, principal component analysis, competing endogenous RNA network, prognostic long non-coding RNA expression signature

## Abstract

Long non-coding RNAs (lncRNAs) are non-protein-coding RNAs longer than 200 nucleotides. Accumulating evidence demonstrates that lncRNA is a potential biomarker for cancer diagnosis and prognosis. However, there are no prognostic biomarkers and lncRNA models for multiple myeloma (MM). Hence, it is necessary to screen novel lncRNA that can potentially participate in the initiation and progression of MM and consequently construct a risk score system for the disease. Raw microarray datasets were obtained from the Gene Expression Omnibus website. Weighted gene co-expression network analysis and principal component analysis identified 12 lncRNAs of interest. Then, univariate, least absolute shrinkage and selection operator Cox regression and multivariate Cox hazard regression analysis identified two lncRNAs (LINC00996 and LINC00525) that were formulated to construct a risk score system to predict survival. Receiver operating characteristic analysis certificated the superior performance in predicting 3-year overall survival (area under the curve = 0.829). The similar prognostic values of the two-lncRNA signature were also observed in the tested The Cancer Genome Atlas dataset. Furthermore, two other lncRNAs (LINC00324 and LINC01128) were differentially expressed between CD138+ plasma cells from normal donors and MM patients and were verified to be associated with cancer stage in the Gene Expression Omnibus dataset. A lncRNA-mediated competing endogenous RNA network, including 2 lncRNAs, 12 mitochondrial RNAs, and 103 target messenger RNAs, was constructed. In conclusion, we developed a two-lncRNA expression signature to predict the prognosis of MM and constructed a key lncRNA-based competing endogenous RNA network in MM. These lncRNAs were associated with survival and are probably involved in the occurrence and progression of MM.

## Introduction

Multiple myeloma (MM) is the second most common hematological malignancy. It is caused by the clonal proliferation of malignant plasma cells in the bone marrow (BM) (Laubach et al., 2011). MM is characterized by renal impairment, lytic bony lesions, anemia, and bone pain. The survival of MM patients ranges from a few weeks to more than 10 years (Decaux et al., 2008; Chen W. C. et al., 2017; Cowan et al., 2018).

As a newly discovered type of non-coding RNA, long non-coding RNAs (lncRNAs) function as imperative regulators involved in tumorigenesis, tumor suppression (Poliseno et al., 2010; Hung and Chang, 2010), and many biological processes (Geisler and Coller, 2013; Fatica and Bozzoni, 2014). Many lncRNAs involved in the initiation and progression of MM have been identified. Furthermore, lncRNAs can also regulate gene expression by interacting with mitochondrial RNA (miRNA) at miRNA-binding sites (MREs). For example, MALAT1 is an lncRNA that inhibits the proliferation and adhesion of myeloma cells by upregulating the expression of miR-181a-5p (Sun et al., 2019a). The aberrant expression of urothelial cancer associated 1 lncRNA affords it the ability to promote proliferation and inhibits apoptosis by regulating miR-1271-5p and hepatocyte growth factor in MM cells (Yang and Chen, 2019). Abnormally expressed lncRNA NR_046683 in patients of different MM subtypes and stages indicated that it could be used as a new indicator for potential drug target and prognosis (Dong et al., 2019). Although several lncRNA prognostic models have been identified in uterine corpus endometrial carcinoma (Ouyang et al., 2019), hepatocellular carcinoma (Sun et al., 2019b), cervical cancer (Wu et al., 2019), and lung adenocarcinoma (Zhou et al., 2019), the clinical implication of most lncRNAs in MM remains unclear.

Weighted gene co-expression network analysis (WGCNA) is an algorithm that is frequently used to cluster highly synergistically altered gene sets into separate modules. This can establish connections with clinical traits and thus screen out candidate indicator genes or therapeutic targets (Langfelder and Horvath, 2008; Shi et al., 2010). Principal component analysis (PCA) is another mathematical algorithm. It is a powerful technique that is widely applied in bioinformatics and other fields. It can reduce the dimensionality of the data while retaining most of the variations that are uncorrelated in the data set. These unrelated variables are called principal components (PCs) (Ringner, 2008). After identifying new variables, the PCs, with a sample-like pattern and a weight for each gene, further exploration can be done by building a link with clinical data, and candidate genes can be obtained by comparing component loadings. In the present study, the Gene Expression Omnibus (GEO) public integrated database provided an application platform of genomic sequencing data along with the clinical information of each MM patient. WGCNA and PCA were performed to explore public sequencing data and clinical information of MM patients.

A few key gene modules associated with tumor stage and PCs correlated with risk score and proliferation index were identified, and 12 lncRNAs in the intersection were identified. We found a two-lncRNA signature that might act as an independent prognostic factor to identify MM patients that are at higher risk of poor clinical outcome. Furthermore, using other datasets, we recognized database of essential genes (DEG) and constructed a competing endogenous RNA (ceRNA) network in MM based on two of the 12 abnormally expressed lncRNAs. These two lncRNAs may participate in tumorigenesis or serve as clinical indicators of the progression of MM.

## Results

### Weighted Gene Co-expression Network Analysis Identification of Clinically Significant Modules

**FIGURE 1:**
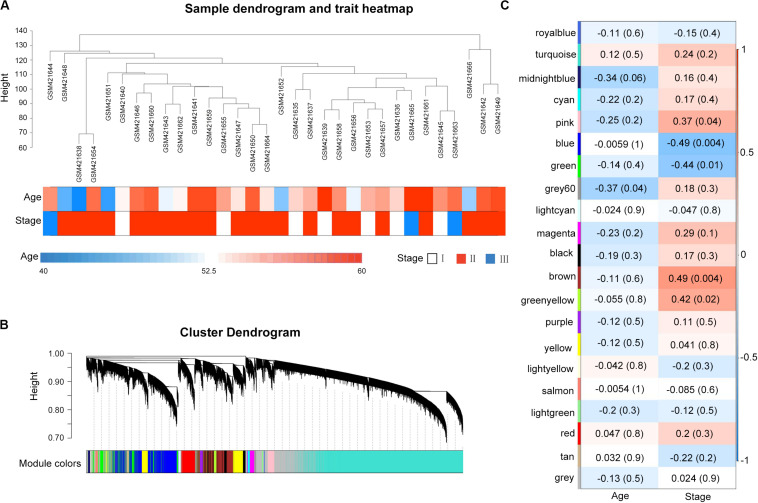
Weighted gene co-expression network of multiple myeloma and module–trait relationships. **(A)** Cluster analysis of samples and clinical traits. All the samples were in the clusters. **(B)** Gene dendrogram obtained by clustering all genes from GSE16791. Each branch in the figure corresponds to one gene, and each color to one co-expression gene module. **(C)** Module clinical associations. Each row represents a module eigengene, and each column represents a clinical trait. Each cell contains the corresponding correlation coefficient and the *P*-value. The blue and green modules were significantly correlated with a stage.

A total of 32 MM samples with a known stage of cancer were utilized to conduct the hierarchical clustering analysis using the WGCNA package. The sample dendrogram and clinical trait heatmap of GSE16791 is displayed in Figure 1A. No obvious outlier was evident in the sample clustering. The information of two clinical traits of 32 MM samples, including age and cancer stage, is presented in Figure 1A. Selecting the best soft-thresholding powers is imperative to obtain relatively balanced scale independence and mean connectivity. As presented in Supplementary Figure S2A, we selected β = 8 (scale-free *R*^2^ = 0.81) as a soft-threshold to construct a scale-free network, and a total of 21 modules were detected (Figure 1B). As the overall gene expression level of the corresponding module, the module eigengenes were calculated to assess the relationship between modules and clinical information by Pearson’s correlation analysis. The results indicated that the stage was negatively associated with blue and green modules (Figure 1C). Scatterplots of gene significance of stage vs. module membership in the blue and green modules revealed that they were highly correlated (Supplementary Figure S2B). Also, we calculated eigengenes of all modules and clustered them on the base of their correlations. A module eigengenes dendrogram indicated that the blue and green modules were clustered together, and the eigengene network heatmap revealed similar results (cor = 0.65, *P* = 5e-05; Supplementary Figure S2C). Therefore, we chose blue and green modules for further analysis.

### Principal Component Analysis Determination of Interesting Principal Components Associated With Clinical Traits

**FIGURE 2:**
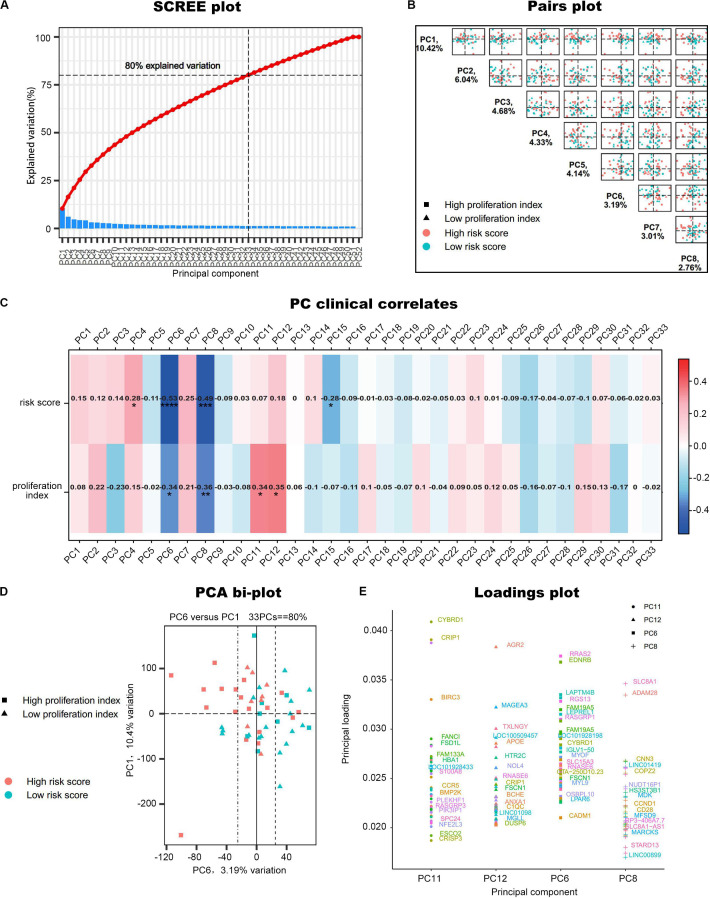
PCA of GSE17306. **(A)** PCs accounted for 80% of the explained variation in the dataset, and the first 33 PCs were responsible for the same. **(B)** A plot comparing PC1–PC8 on a pairwise basis. PC1 is usually the most important part of PCA. **(C)** Correlation of the principal components (PCs) to the clinical data. *P < 0.05, **P < 0.01, ***P < 0.001, ****P < 0.0001. **(D)** A bi-plot of PC1 versus PC6. **(E)** Determine the variables that drive variation among each PC. Components have a sample-like pattern with a weight called component loading for each gene. Genes ranked the top 20 of the loadings range were presented.

Principal component analysis was performed on the 52 samples in GSE17306. In this dataset, the gene expression profiling (GEP)-risk score and proliferation index of each sample were calculated according to the GEP (Zhou et al., 2010). Initially, PCA created 52 composite variables (PCs) by reducing the dimensionality of numerous genes. The first 33 components, which explained 80% of the variability among the 52 samples, were retained to correlate clinical traits (Figure 2A). These 33 composite variables are enough to explain the sample differences to the greatest extent. Next, to ascertain the capability of PCs to differentiate risk score level and proliferation index level, the pairs plot was conducted to compare PC1 with PC8 on a pairwise basis (Figure 2B). Additionally, a bi-plot of PC1 versus PC6 indicated that PC6 could roughly distinguish the high-risk group from the low-risk group (Figure 2D). Next, we correlated the PCs back to the clinical data, including the GEP-risk score and proliferation index, to identify interesting PCs. PC6 and PC8 were negatively associated with risk score and proliferation index in all the 33 PCs retained (Figure 2C). PC11 and PC12 were positively correlated with the proliferation index. For each PC of interest, “plotloadings” determined the genes ranked in the top 20 of the loadings range and then created a final consensus list of these (Figure 2E).

### Construction a Risk Assessment Model

**FIGURE 3:**
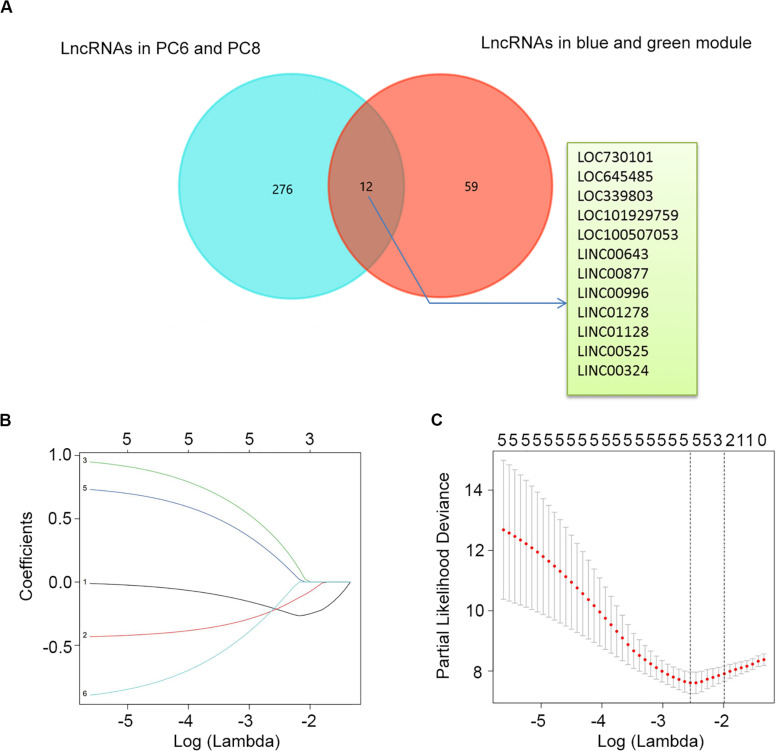
Determination of candidate lncRNAs and LASSO regression analysis. **(A)** Venn diagram of candidate lncRNAs in blue and green modules and PC6 and PC8. **(B)** LASSO coefficient profiles of the six candidate lncRNAs. **(C)** Ten-fold cross-validation used to tune parameter selection in the LASSO model. A vertical line is drawn at the value chosen by 10-fold cross-validation.

**FIGURE 4:**
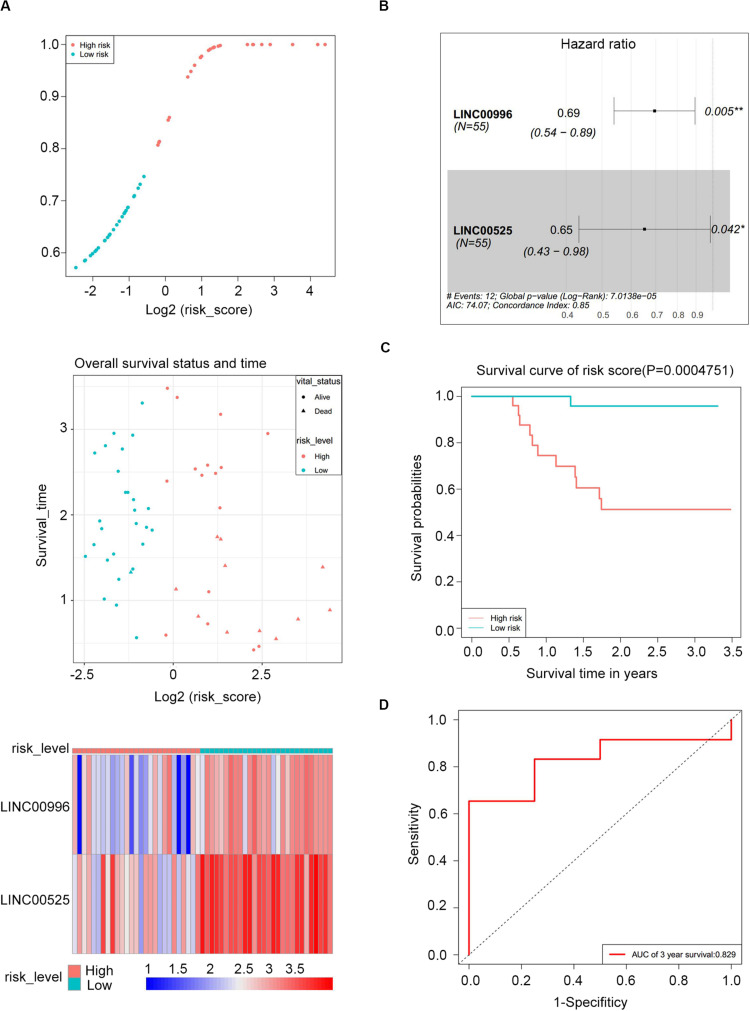
Risk score performance in the GSE57317 (training) datasets. **(A)** Risk score of the two lncRNAs in 55 MM patients (top); overall survival status and duration (middle); heatmap of the two lncRNA expression in MM patients (bottom). **(B)** Forest plot showing the hazard ratios with 95% confidence interval of the multivariate Cox regression results. **(C)** Overall survival of the high- and low-risk score groups. **(D)** Three-year survival receiving operating characteristic curve (ROC) according to the two-lncRNA signature risk score (red).

To construct a lncRNA scoring system that is predictive of survival in the MM patients, we extracted lncRNAs from the blue and green modules and PC6 and PC8 based on the Genecode annotation^1^. Finally, a total of 12 lncRNAs were obtained from the intersection of the interesting modules and PCs (Figure 3A). The expression levels of 12 lncRNAs were extracted from GSE57317 to conduct the univariate Cox regression analysis. The results of the univariate Cox analysis of 12 prognostic lncRNAs from the discovery cohort are shown in Table 1. After this, six significant lncRNAs (*P* < 0.05) were identified and were included in the least absolute shrinkage and selection operator (LASSO) model; cross-validation was adopted to select the penalty parameters (Figures 3B,C). Two lncRNAs were identified based on lambda.1se values (Supplementary Table S1). The quantitative real-time polymerase chain reaction (qRT-PCR) results showed that the expression of LINC00525 was significantly downregulated in Roswell Park Memorial Institute (RPMI)-8226 and KM3 cell lines, whereas LINC00996 was significantly upregulated in KM3 cell line compared with normal plasma cells (Supplementary Figures S3A,B). We further included expression levels of the two lncRNAs in a multivariate Cox model. The risk score = (−0.3647) × (expression value of LINC00996) + (−0.4266) × (expression value of LINC00525). The details of the two lncRNAs are depicted in Figure 4B. We used the median of the risk score as the cutoff to define the groups of MM patients with high and low scores (Figure 4A). The survival time and overall survival (OS) status in the training dataset are presented in the middle panel of Figure 4A. Compared with those in the low-risk score group, patients in the high-risk score group displayed an obviously worse OS (Figure 4C). The 3-year survival receiver operating characteristic (ROC) curve was also plotted. The area under the curve of the risk score reached 0.829 (Figure 4D),revealing that the risk score based on the two lncRNAs is a good indicator of prognosis. The results of univariate and multivariate Cox regression analyses indicated that the risk score (*P* < 0.001 and *P* = 0.006) was an independent prognostic indicator (Supplementary Table S2). To further examine the accuracy of the lncRNA risk score model developed in the training dataset, the performance of the risk score was also evaluated in The Cancer Genome Atlas (TCGA) dataset. The result of multivariate Cox regression analysis for the expression level of two lncRNAs in the TCGA dataset is presented in Supplementary Figure S4B. The risk survival status, score distribution, and expression pattern of the two lncRNAs in the 787 MM patients in the TCGA dataset are displayed in Supplementary Figure S4A. Also, corresponding to our previous conclusion, the OS was significantly shorter in the high-risk group compared with that in the low-risk group (Supplementary Figure S4C), and the AUC of the risk score reached 0.584 (Supplementary Figure S4D). Univariate Cox regression analyses were conducted to detect various factors correlated with prognosis. The results revealed that age (*P* = 0.009), tumor stage (*P* < 0.001), and risk score (*P* = 0.002) were significantly associated with the OS of the MM patients. A subsequent multivariate Cox regression analysis indicated that the tumor stage (*P* < 0.001) and risk score (*P* = 0.001) were independent prognostic indicators (Supplementary Table S3).

**TABLE 1:** Univariate Cox analysis of 12 prognostic lncRNAs from the discovery cohort.

lncRNA name	Type	HR	*P*
LINC01128	Bad	5.324513	0.015396
LOC339803	Bad	2.530873	0.031322
LOC100507053	Bad	1.474999	0.259866
LINC01278	Bad	1.437495	0.173067
LINC00643	Bad	1.097326	0.578361
LINC00877	Good	0.876417	0.512653
LOC645485	Good	0.85804	0.474318
LINC00996	Good	0.602728	7.11E-05
LINC00525	Good	0.575173	0.001695
LOC101929759	Good	0.464721	0.040177
LINC00324	Good	0.382086	0.004307

*HR, hazard ratio. Type represents bad survival lncRNAs and good survival lncRNAs. All statistical tests were two-sided.*

### Detection and Validation of Differentially Expressed Long Non-coding RNAs

**FIGURE 5:**
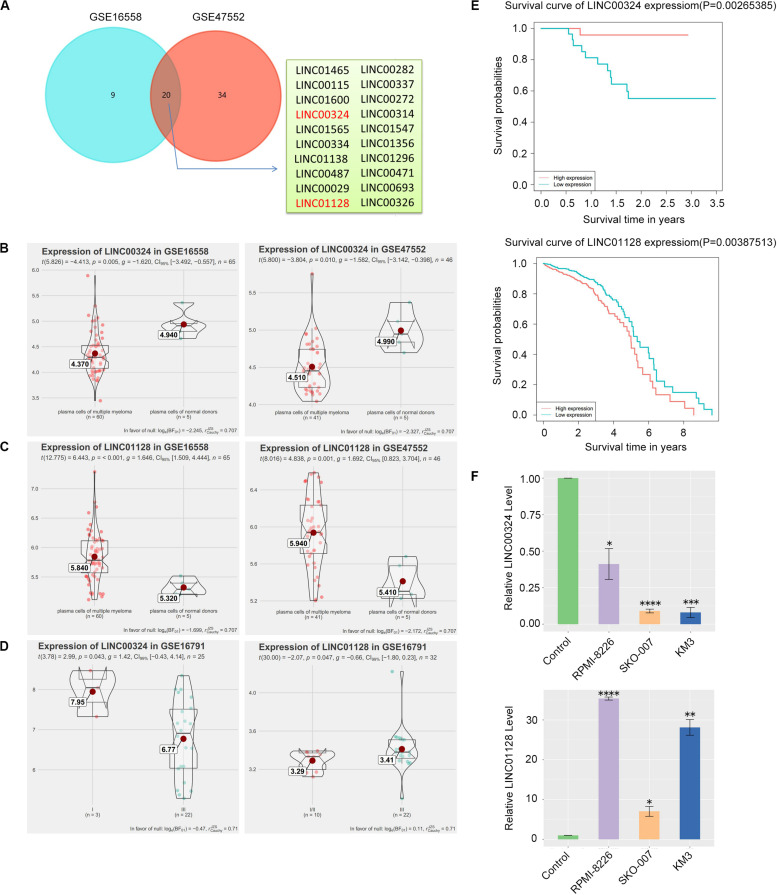
Detection and validation of differentially expressed lncRNAs (DELs). **(A)** Venn plot of DELs between GSE16558 and GSE47552. **(B)** GSE16558 and GSE47552 indicated the lower expression of LINC00324 in CD138+ plasma cells of MM patients compared with normal donors. **(C)** GSE16558 and GSE47552 indicated the higher expression of LINC01128 in CD138+ plasma cells of MM patients compared with normal donors. **(D)** Relationship between the two lncRNAs and cancer stages in GSE16791. **(E)** GSE57317 dataset (left) and TCCA dataset (right) revealed that MM patients with low expression of LINC00324 and high expression of LINC01128 had an obviously poorer overall survival. **(F)** Expression of LINC00324 (top) and LINC01128 (bottom) in human multiple myeloma cell lines (RPMI-8226, SKO-007, KM3) as well as normal plasma cells. Data are presented as the mean ± standard deviation. *P < 0.05, **P < 0.01, ***P < 0.001, ****P < 0.0001.

CD138+ plasma cells obtained from healthy donors in GSE16558 and GSE47552 were analyzed. Based on the cutoff criteria of *P* < 0.05, 20 DELs were detected (Figure 5A). Surprisingly, among the 12 prognostic lncRNAs we identified earlier, LINC00324 and LINC01128 are abnormally expressed (Figures 5B,C). The relationship between the two lncRNAs and cancer stages in GSE16791 is displayed in Figure 5D. Expression levels of the two lncRNAs among patients with different stages were compared, and statistical differences were calculated using Student’s *t*-test. Corresponding to our previous WGCNA and PCA results, patients with poorly differentiated stage III cancer displayed significantly lower LINC00324 expression levels compared with patients with moderately differentiated cancer of less advanced stage I. Furthermore, increased expression of LINC01128 was correlated with advanced MM stage. Also, to determine the prognostic value of these two lncRNAs in MM, the survival data of MM patients were obtained from the TCGA database and GSE57317. As presented in Figure 5E, patients with high LINC01128 expression exhibited a significantly poorer OS rate compared with patients with high LINC01128 expression. On the contrary, we observed that patients with higher LINC00324 expression had better OS than those with lower LINC00324 expression. These results indicate that LINC00324 may be a tumor suppressor gene, whereas LINC01128 may be a cancer gene. The qRT-PCR results also showed that the expression pattern of the two lncRNAs in MM cells and normal plasma cells was similar to the microarray results (Figure 5F). LINC01128 was upregulated, whereas LINC00324 was significantly downregulated in the three MM cell lines.

### Co-expression Network of Key Long Non-coding RNAs and Differentially Expressed Messenger RNAs in the Blue Module

**FIGURE 6:**
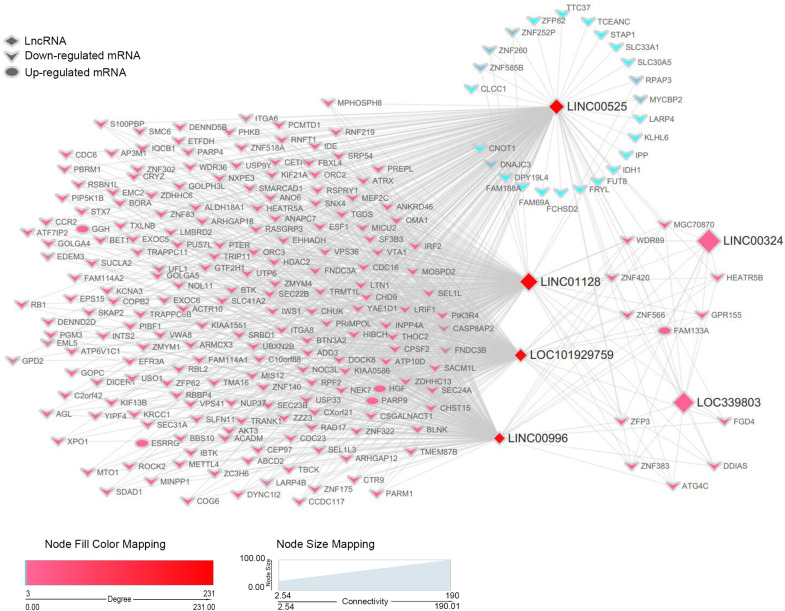
A co-expression network of six lncRNAs and DEmRNAs in the blue module. For simplicity, only DEmRNAs with a connection with the interesting lncRNAs in the blue module were retained to construct the co-expression subnetwork. lncRNAs are depicted by diamonds, whereas DEmRNAs are indicated by the rounded rectangles (upregulation) or vs. (downregulation). Size of genes is related to the intra-modular connectivity, and the color is related to the weighted score of the interactions.

Based on the previous results, we recognized six lncRNAs (LINC00525, LINC00996, LINC01128, LINC00324, LINC101929759, and LINC339803) as potential biomarkers or prognostic indicators. These lncRNAs were all in the blue module. To further dissect the role of six lncRNAs in MM, we created a gene co-expression subnetwork for the genes in the blue module according to their topology overlap matrix similarity; messenger RNAs (mRNAs) connected to six lncRNAs are too much to display perfectly; thus, we selected only differentially expressed mRNAs (DEmRNAs) to construct a network. Our lncRNAs may potentially regulate these co-expressed DEmRNAs through the ceRNA mechanism. DEmRNAs were obtained from GSE16558 and GSE47552 based on the cutoff criteria of a *P*-value < 0.05; | log (FC)| > 1.680 DEmRNAs that overlapped in GSE16558 and GSE47552 were identified (Supplementary Table S4). Finally, the connections between the six lncRNAs and DEmRNAs are displayed in Figure 6. LncRNAs are shown by diamonds, whereas DEmRNAs are represented by round rectangles (upregulation) or vs. (downregulation). The size of the nodes reflects the strength of connectivity, and the color is related to the weighted score of the interactions.

### Functional Annotation

**FIGURE 7:**
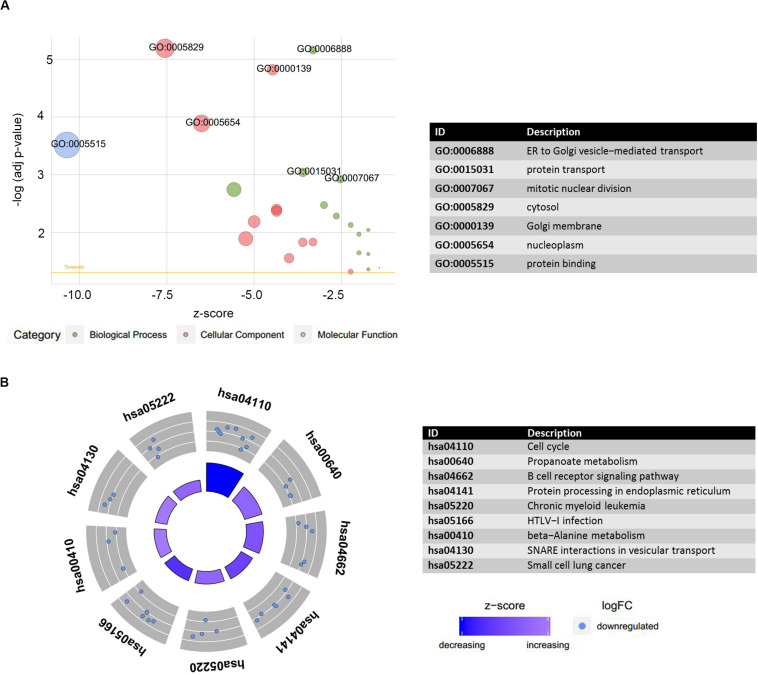
Functional annotation analysis of DEmRNAs co-expressed with LINC01128. **(A)** Gene ontology (GO) terms enrichment analysis was performed using the Database for Annotation, Visualization, and Integrated Discovery and visualized by GOplot. Significance of a term is indicated by the adjusted *P*-value (adj_p-val). logFC of selected genes is taken from GSE16558. *z*-score provided a hint if the biological process (/molecular function/cellular components) was more likely to be decreased (negative value) or increased (positive value). *z*-score is assigned to the *x*-axis and the negative logarithm of the adjusted *P*-value to the *y*-axis. Area of the displayed circles is proportional to the number of genes assigned to the term, and the color corresponds to the category. A threshold for the labeling is set as log(adj_p-value) > 2.8. **(B)** Plot of the enriched KEGG pathway. Outer circle shows a scatter plot for each term of the logFC of the assigned genes. Red circles display upregulation and blue ones downregulation by default. There were no upregulated DEmRNAs co-expressed with LINC01128 enriched.

The preceding findings indicated that the LINC00324 and LINC01128 were potentially involved in the occurrence and progression of MM. To more precisely understand the biological relevance and function of these two lncRNAs, we uploaded DEmRNAs, which were co-expressed with key lncRNAs in the blue module into the Database for Annotation, Visualization, and Integrated Discovery to conduct Gene Ontology (GO) and Kyoto Encyclopedia of Genes and Genomes (KEGG) analyses. The results were visualized using the GOplot R package. The results of the differential analysis were used to calculate a *z*-score for presenting enriched KEGG pathways (Supplementary Table S4). Regarding enriched GO terms, DEmRNAs co-expressed with LINC01128 were mainly enriched in the endoplasmic reticulum to Golgi vesicle-mediated transport, protein transport, mitotic nuclear division, cytosol, Golgi membrane, nucleoplasm, and protein binding (Figure 7A). Regarding the enriched KEGG pathways, there were no upregulated DEmRNAs co-expressed with LINC00324 enriched, and other downregulated DEmRNAs were significantly enriched in the cell cycle, propanoate metabolism, B-cell receptor signaling pathway, protein processing in the endoplasmic reticulum, chronic myeloid leukemia, human T-cell lymphotropic virus type 1 infection, and beta-alanine metabolism (Figure 7B). There were no significant results for LINC00324 because too few mRNAs are connected with it.

### Gene Set Enrichment Analysis and Gene Set Variation Analysis Reveal a Close Relationship Between Key Long Non-coding RNAs, Multiple Cancer-Related Pathways, and Metabolic Pathways

**FIGURE 8:**
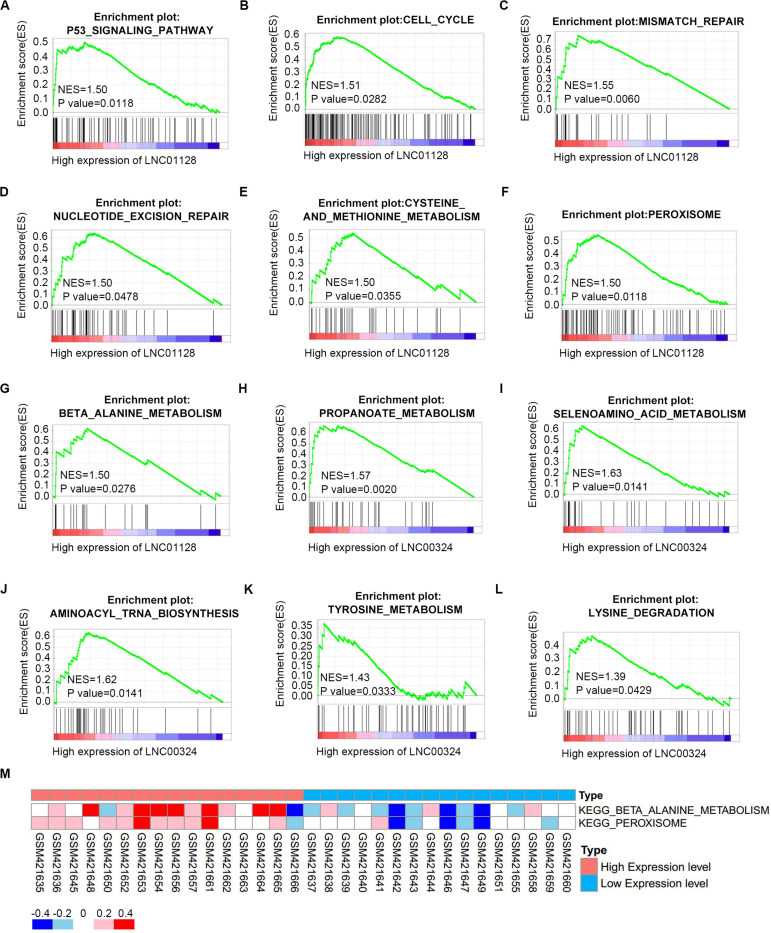
Gene set enrichment analysis (GSEA) and gene set variation analysis (GSVA) of two lncRNAs in GSE16791. **(A–G)** GSEA results of c2 reference gene sets for high LINC01128 expression groups in GSE16791. **(H–L)** GSEA results of c2 reference gene sets for high LINC00324 expression groups in GSE16791. **(M)** GSVA-derived clustering heatmaps of differentially expressed pathways for LINC01128 in GSE16791.

To further investigate the potential functions of LINC01128 and LINC00324, we performed gene set enrichment analysis (GSEA) and gene set variation analysis (GSVA) on the GSE16791 dataset. We divided these samples into two groups based on the expression levels of these two lncRNAs. As shown in Figures 8A–G, samples in GSE16791 with high expression of LINC01128 were enriched in multiple cancer-related pathways, including the P53 signaling pathway, cell cycle, mismatch repair, nucleotide excision repair, and several metabolic pathways, including cysteine and methionine metabolism, peroxisome, and beta-alanine metabolism. Also, our previous finding that the DEmRNAs co-expressed with LINC01128 were enriched in beta-alanine metabolism was, surprisingly, verified by GSEA and GSVA results (Figures 8G,M). The expression level of LINC00324 was also extracted for enrichment analysis. Genes in the high expression groups of LINC00324 were mainly involved in multiple metabolic pathways, including propanoate metabolism, selenoamino acid metabolism, aminoacyl-tRNA biosynthesis, tyrosine metabolism, and lysine degradation (Figures 8H–L).

### Long Non-coding RNA-Mediated Competing Endogenous RNA Network Revealed Potential Mechanisms of LINC01128 and LINC00324

**FIGURE 9:**
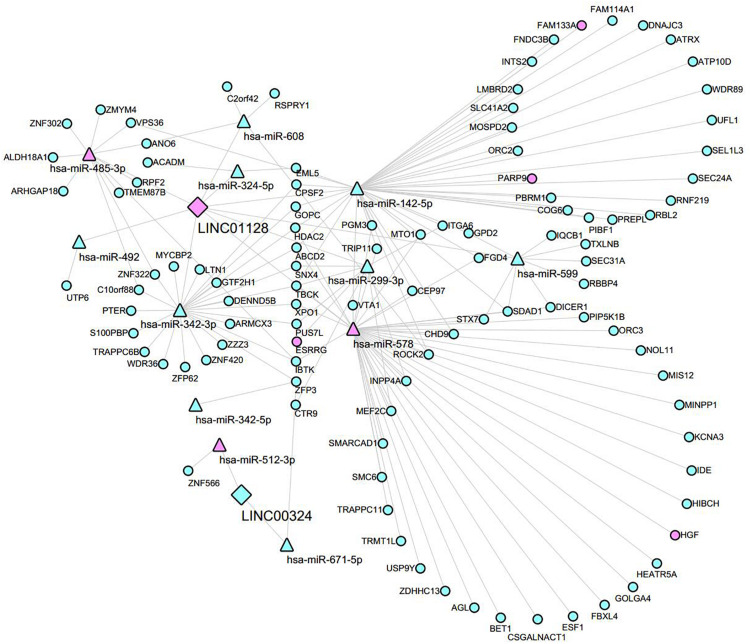
Global view of the ceRNA network in MM. Network consists of two lncRNA nodes, 12 miRNA nodes, and 103 mRNA nodes. Diamonds indicate lncRNAs, triangles indicate miRNA, and ellipses indicate mRNA. Pink and blue represent up- and downregulation, respectively.

To investigate the interaction between the lncRNA and mRNAs, the lncRNA–miRNA–mRNA network was constructed according to the ceRNA hypothesis by integrating expression profile data and their regulatory relationships. We obtained DEmRNAs, DEmiRNAs based on the criteria mentioned in section “Materials and Methods.” The interaction between the two lncRNAs and miRNAs were first predicted through Starbase3.0 and the RNA22 tool. We then predicted that the potential DEmiRNAs can target LINC01128 and LINC00324 co-expressed DEmRNAs in the blue module using DIANA TOOLS (Supplementary Table S5). Finally, a total of 12 miRNAs overlapped in our prediction results; 2 lncRNAs and 103 mRNAs were included in the ceRNA network (Supplementary Table S6), and their regulatory relationships were visualized by Cytoscape (Figure 9). In this network, different shapes represent different RNA types, with pink and blue denoting up- and downregulation, respectively.

## Discussion

Multiple myeloma is the most common primary bone cancer among 70-year-old and older American adults (Reisenbuckler, 2014). Although genetic and epigenetic events contributing to the occurrence and progression of MM have been increasingly identified, the diagnosis, treatment, and clinical outcome of MM remain mostly unclear (Prideaux et al., 2014). More recently, aberrant lncRNA expression in MM was observed and further validated to be involved in epigenetic, transcriptional, and posttranscriptional regulation (Meng et al., 2018). Several lncRNA prognostic models have been identified in multiple cancers, including hepatocellular carcinoma (Zhang et al., 2020), bladder cancer (Zhou et al., 2020), non-small cell lung cancer (Zhou et al., 2015a; Sun et al., 2020), breast cancer (Shen et al., 2020), glioma (Wang et al., 2018), glioblastoma (Zhou et al., 2018), and diffuse large B-cell lymphoma (Zhou et al., 2017). These studies had highlighted the diagnostic and prognostic roles of lncRNAs, and the lncRNA signatures they constructed had an imperative value for survival predicting for different cancer patients. Therefore, identifying new and effective prognostic biomarkers and establishing a reliable prognostic model based on lncRNA expression signature are critical for patients with MM.

WGCNA is a powerful algorithm that has not yet been utilized to analyze the expression profile of MM samples. Presently, a total of 32,216 genes, which were not all DEGs, were selected to conduct WGCNA analysis in case of missing significant information. Furthermore, we applied PCA for the first time to correlate PCs with clinical traits to find key lncRNAs. Then, 12 key lncRNAs that were associated with cancer stage, risk score, and proliferation index were identified in the intersection of key modules and PCs. Univariate Cox regression analysis retained six significant lncRNAs (*P* < 0.05) for further analysis. A co-expression network of six lncRNAs and co-expressed DEmRNAs in the blue module was constructed to present the co-expression pattern and the relationship between key lncRNAs and DEmRNAs. This network can provide insights for identifying possible targets of key lncRNAs. After the LASSO and Cox proportional hazard regression analysis, we detected a prognostic formula for predicting survival based on the two lncRNAs, including LINC00525 and LINC00996, and verified it in the testing set. The patients were ultimately divided into high- or low-risk patients according to the median risk value. Kaplan–Meier analysis showed that the patients in the high-risk score group displayed obviously worse OS compared with those in the low-risk score group. Furthermore, ROC curve analysis revealed the stability and accuracy of the two-lncRNA signature in predicting patient prognosis. Further analysis showed that the two-lncRNA risk score signature is an independent predictor of MM patient prognosis. Indeed, prior studies had established several lncRNA prognostic signatures that can provide a comprehensive clinical assessment of MM prognosis (Zhou et al., 2015b; Samur et al., 2018). Significantly, instead of simply utilizing survival associated lncRNAs to construct lncRNA prognostic signatures, it Is our first time to combine WGCNA and PCA to select prognostic lncRNAs that could be further used to establish a survival model. Subsequently, we performed a series of rigorous analyses, including univariate, LASSO Cox regression, and multivariate Cox hazard regression analysis to realize exact survival prediction. Additionally, in contrast to the earlier lncRNA model in MM (Zhu et al., 2020), we utilized an external dataset to examine the accuracy of our lncRNA signature.

Many recent studies have indicated that lncRNAs can regulate gene expression by interacting with the miRNA via MREs in MM (Sun et al., 2019a; Yang and Chen, 2019). Thus, it is imperative to recognize MM-specific lncRNAs as biomarkers and determine their potential mechanisms. These lncRNAs may be essential in the initiation and development of MM. Firstly, we identified 12 interesting lncRNAs, which may participate in the development of MM. To further select MM-specific lncRNAs, we screened DElncRNAs that overlapped in GSE16558 and GSE47552. Surprisingly, our PCR and microarray results indicated that 2 of the 12 lncRNAs (LINC01128 and LINC00324) were differentially expressed. LINC00324 can promote proliferation and metastasis but can inhibit cell apoptosis of lung adenocarcinoma cells by sponging miR-615-5p to promote AKT1 expression (Pan et al., 2018). Similar results were also found where LINC00324 can promote gastric cancer cell proliferation by binding with HuR and stabilizing FAM83B expression (Zou et al., 2018). It can also be used to predict the prognosis in patients with thymoma (Gong et al., 2018). There are no references for LINC01128. Its potential function remains to be determined. Next, GO analysis revealed that those LINC01128 co-expressed DEmRNAs were associated with protein transport and protein binding processes. KEGG pathway analysis demonstrated that they were enriched in cancer-related pathways, including cell cycle, chronic myeloid leukemia, small cell lung cancer, and metabolism-related pathways, including propanoate metabolism and beta-alanine metabolism. To further explore the underlying mechanism of LINC00324 and LINC01128, we formulated a ceRNA network based on predicted interactions between DEmiRNAs and DEmRNAs. Based on our network and the ceRNA mechanism, we speculated that LINC01128 might act as a tumor suppressor in MM through multiple mechanisms, including miR-142-5p/PARP9 or FAM133A axis, and the miR-299-3p/estrogen-related receptor gamma axis. The cancer–testis antigen FAM133A is a downstream target of miR-155 and is a negative regulator of glioma invasion and migration (Huang et al., 2018). Estrogen-related receptor gamma is a tumor suppressor as well as an activator of multiple cancers, including gastric cancer (Kang et al., 2018), breast cancer (Kumari et al., 2018), laryngeal squamous cell carcinoma (Shen et al., 2019), and liver cancer (Kim et al., 2016). LINC00324 may exert tumor-promoting functions in MM through targeting the miR-512-3p/ZNF566 axis. However, this remains to be verified. Finally, GSEA revealed that samples with high expression of LINC01128 were in multiple cancer-related pathways, including the P53 signaling pathway, cell cycle, mismatch repair, nucleotide excision repair, and several metabolic pathways, including cysteine and methionine metabolism, peroxisome, and beta-alanine metabolism. Several studies have reported that the cell cycle, P53 signaling, and DNA repair-related pathways are important tumor biological mechanisms (Balint and Vousden, 2001; Jackson and Bartek, 2009). Also, high beta-alanine concentrations are linked with cancer (Pine et al., 1982; Nishimura et al., 2012). Our findings suggested that the high expression of LINC01128 may be crucial in tumorigenesis and progression of MM, probably by regulating the cell cycle, DNA damage, or amino acid metabolism. Corresponding with our predicted mechanism of LINC01128, the mutation of the NAD+ binding site in PARP9 has been reported to increase the DNA repair activity of the heterodimer (Yang et al., 2017). On the other hand, genes in high expression groups of LINC00324 were mainly involved in multiple metabolic pathways, including propanoate metabolism, selenoamino acid metabolism, aminoacyl-tRNA biosynthesis, tyrosine metabolism, and lysine degradation. These observations can be explained by the hypothesis that LINC00324 suppresses tumorigenesis of MM by interfering with carbohydrate metabolism, amino acid metabolism, and protein translation.

In conclusion, WGCNA and PCA were performed to correlate the gene expression profile of patients with MM to the corresponding clinical traits. We identified lncRNAs that may potentially be involved in the initiation and development of MM. Finally, a two-lncRNA risk score model was formulated, and its precise prediction value was demonstrated. We also identified two lncRNAs as biomarkers and predicted their possible function as ceRNAs. These findings provide fundamental insights for further basic studies.

## Materials and Methods

### Gene Expression Profile Data and Clinical Characteristics

The overall design and workflow of this study are presented in Supplementary Figure S1. The RNA expression profiles of MM patients and normal donors were identified from the GEO database^2^ (Table 2). GSE16791 was utilized to conduct a WGCNA analysis for this study. This series of microarray experiments include 16,325 mRNA and 1,137 lncRNA expression profiles of purified plasma cells (PCs) obtained from 32 newly diagnosed MM. GSE17306 is a microarray analysis that contains 16,401 mRNA, 556 miRNA, and 1,146 lncRNA expression profiles of MM patients with corresponding clinical information, including mRNA-based GEP-risk score and proliferation index (Shaughnessy et al., 2007). It was used here to implement the PCA algorithm to correlate clinical traits with gene expression patterns. Corresponding clinical information, including survival time and vital status, was obtained from the GSE57317, including 16,325 mRNA and 1,137 lncRNA expression profiles of 55 MM patients, and TCGA RNA-Seq dataset contains 56,753 mRNA, 1,881 miRNA, and 14,142 lncRNA expression profiles of 765 MM patients to construct lncRNA risk score system. GSE16558, including 18,966 mRNA, 382 miRNA, and 431 lncRNA expression profiles of 60 MM patients and 5 healthy donors, GSE47552, including 18,966 mRNA and 431 lncRNA expression profiles of 41 MM patients and 5 healthy donors, and GSE17498, including 722 miRNA expression profiles of 40 MM patients and 3 healthy donors, were used to screen DEGs including DElncRNAs, DEmiRNAs, and DEmRNAs. Microarray annotation information was utilized to match probes with corresponding genes, and lncRNA expression was obtained based on the annotation of Genecode (see footnote 1).

**TABLE 2:** Summary of included datasets.

Dataset ID	Sample size		Age (year)	Gender		Tumor stage			Vital status	
	Multiple myeloma	Normal		Male	Female	I	II	III	Alive	Dead
GSE16791	32	0	40–65	–	–	3	7	22	–	–
GSE17306	52	2	–	–	–	–	–	–	–	–
GSE57317	55	0	–	–	–	–	–	–	43	12
GSE16558	60	5	–	–	–	–	–	–	–	–
GSE17498	40	3	39–85	23	17					
GSE47552	41	5	–	–	–	–	–	–	–	–
TCGA	765	0	27–88	449	316	266	276	223	609	156

### Weighted Co-expression Network Analysis

A total of 32,216 genes identified in each sample of GSE16791 were utilized to construct a gene co-expression network using the WGCNA R package (Langfelder and Horvath, 2008; Chen L. et al., 2017). Sample clustering of all genes was applied to check if they were good genes and good samples. A scale-free co-expression network was achieved when the soft-threshold power was set as 8 (scale-free *R*^2^ = 0.81), cut height as 0.25, and minimal module size as 30. Then, to evaluate co-expression levels between genes, Pearson correlations were performed and then weighted by raising their absolute value to a power. Hierarchical clustering dendrograms visualized gene modules in different colors. Modules with the highest correlation with cancer stage were selected for further analysis.

### Principal Component Analysis

Principal component analysis compresses all the original variables into a smaller subset of composite variables (PCs) instead of ignoring or discarding variables. PCA tools, a useful R package that provides functions for data exploration, were applied to analyze GSE17306 dataset^3^. At first, PCA helped us to determine PCs, accounting for 80% of the explained variation. Secondly, we correlated the PCs back to the clinical data, including mRNA-based GEP-risk score and proliferation index, to gain interesting PCs. Finally, the plotLoadings function could contribute to determining the variables ranked top 5% of the loadings range.

### Identification and Evaluation of a Risk Assessment Model

The prognostic value of 12 lncRNAs in the intersection of blue and green modules, and PC6 and PC8, were evaluated by a univariate Cox model with a statistical level of significance set at *P* < 00.05. Critical prognostic lncRNAs were further identified by the LASSO regression method (Gao et al., 2010). LASSO regression is a penalized regression method that is often used in machine learning to select the subset of variables. The R glmnet software package was adopted to carry out the LASSO Cox analysis (Tibshirani, 1997). Also, lncRNAs obtained in these steps were then enrolled into a multivariate Cox regression model using a survival R package, and prognosis-associated lncRNAs were selected. The risk score of each patient was calculated based on the summation of each lncRNA and its coefficient, and we distinguished high- from low-risk patients according to the median risk score. The Kaplan–Meier method was applied to analyze the difference of OS between two groups, and a ROC analysis was adopted to estimate the predictive power of this lncRNA risk score system. The TCGA dataset served as a testing set for further validation.

### Construction of Co-expression Network of Key Long Non-coding RNAs and Differentially Expressed Messenger RNAs in the Blue Module

The multivariate Cox regression analysis identified six lncRNAs with *P* < 0.05, which were considered as key lncRNAs. We created a gene co-expression subnetwork for the genes in the blue module according to their topology overlap matrix similarity; DEmRNAs connected to key lncRNAs were selected to construct a co-expression network using Cytoscape. DEmRNAs that overlapped in GSE16558 and GSE47552 (*n* = 680) were identified based on the cutoff criteria of *P* < 0.05 and | log (FC)| > 1. The size of the nodes reflected the strength of connectivity, and the color was related to the weighted score of the interactions.

### Screening of Database of Essential Genes and Survival Analysis

The Limma package in R (Ritchie et al., 2015) was used to identify the DEGs from GSE16558 and GSE47552. We identified DElncRNAs and DEmiRNAs according to the criterion that adjusted *P* < 0.05. Abnormally expressed miRNAs in GSE17306, GSE16558, and GSE17498 were all selected for constructing the ceRNA network. The two DElncRNAs were utilized to perform Kaplan–Meier analysis and log-rank test to identify whether they were correlated with OS using the GSE57317 and TCGA datasets. Log-rank test with *P* < 0.05 was set as statically significant.

### Cell Lines and Clinical Specimens

The RPMI-8226, SKO-007, and KM3 MM cell lines were a generous gift of Prof. Yumin Huang, Department of Hematology, First Affiliated Hospital of Zhengzhou University. Cells were maintained in RPMI-1640 medium (Sigma-Aldrich, St. Louis, MO, United States) with 10% fetal bovine serum at 37°C in an atmosphere of 5% CO_2_. BM was obtained from three healthy controls from a pool of volunteers without any diseases. All volunteers provided written informed consent, and the research ethics committee of the First Affiliated Hospital of Zhengzhou University approved the study (2019-KY-357). Flow cytometry was performed using the CD138 antibody (PE, BD Bioscience, United States) to isolate CD138-positive PC from BM samples according to the manufacturer’s protocol.

### Quantitative Real-Time Polymerase Chain Reaction

Total RNA was extracted using TRIzol reagent (Invitrogen, Carlsbad, CA, United States). A NanoDrop 2000 spectrophotometer (Thermo Fisher Scientific, Waltham, MA, United States) was utilized to detect RNA purity and concentration. RT-PCR was performed using a FastStart Universal SYBR Green Master (Servicebio, Wuhan, China) Kit. Actin was used as an internal control. Primers were synthesized by Servicebio (Wuhan, China). Primer sequences were: LINC01128: Forward 5′-AGGACATAGGCCAGCCAGTAC-3′, Reverse 5′-GTCTTTGGTCCCAGATCACTCC-3′; LINC00324: Forward 5′-ACCTACGGTTTCTGGTCAGCG-3′, Reverse 5′-GACGACGGCAGCCATTACTTT-3′; ACTIN: Forward 5′-CACCCAGCACAATGAAGATCAAGAT-3′, Reverse 5′-CCAGTTTTTAAATCCTGAGTCAAGC-3′. LINC00525: Forward 5′-GCTTTGGAAACTTACTCAGGGTG-3′, Reverse 5′-CTTGAGGCACCAGTGCAAATAC-3′; LINC00996: Forward 5′-GAGGGCACTTTGTCTTACTTGGC-3′, Reverse 5′-ATTCTTCATGCCAATCCTCTCAC-3′. Relative expression was calculated using the 2-△△Ct method. Student’s t-test was conducted by SPSS 25.0 software (SPSS Inc., Chicago, IL, United States) to determine the significance of the differences in mean values.

### Construction of Interesting Differentially Expressed Long Non-coding RNA-Based Competing Endogenous RNA Network

The ceRNA hypothesis posits that lncRNAs can regulate gene expression by interacting with miRNA at miRNA-binding sites (MREs). It is vital to match the DEmRNAs, miRNAs, and lncRNAs to figure out a novel molecular mechanism involved in the development of MM. The MIRanda database^4^, Starbase3.0^5^, and RNA22 tool^6^ were used to predict the interactions between DElncRNAs and miRNAs. The miRNAs that potentially target DEmRNAs were predicted by DIANA Tools^7^. DElncRNAs, DEmRNAs, and DEmiRNAs that overlapped with the predicted miRNAs were selected to construct a ceRNA network and were visualized with Cytoscape version 3.6.1.

### Functional Annotation of Long Non-coding RNA Target Genes

The GO and KEGG enrichment analyses for DEmRNAs, which were co-expressed with LINC00324 and LINC01128, were analyzed using the Database for Annotation, Visualization, and Integrated Discovery database (Huang et al., 2007) and visualized by the GOplot R package (Walter et al., 2015). The *z*-score is a value that can be easily calculated and reveals whether the biological process (molecular function/cellular components) is more likely to be decreased (negative value) or increased (positive value). It is calculated as *z*-score = (up-down)/√count. Up or down represents the number of upregulated or downregulated genes, respectively. The count represents the number of genes that belong to each term. A threshold for the labeling is set as log (adjust *p*-value) > 2.8.

### Gene Set Enrichment Analysis and Gene Set Variation Analysis

The GSE16791 dataset was used to conduct GSEA according to expression levels of two lncRNAs (high expression vs. low expression) (Subramanian et al., 2005). Annotated gene sets c2.cp.kegg. v 7.0.symbols.gmt was chosen as the reference gene sets^8^. The nominal *P*-value estimates the statistical significance of the enrichment score, and a nominal *P*-value ≤ 0.05 was set as the cutoff criterion.

## Data Availability Statement

The datasets GSE16791, GSE17306, GSE57317, GSE16558, and GSE47552 for this study can be found in the GEO database (https://www.ncbi.nlm.nih.gov/geo/). The TCGA datasets were downloaded from TCGA (https://cancergenome.nih.gov/) database.

## Ethics Statement

The studies involving human participants were reviewed and approved by the ethics committee of the First Affiliated Hospital of Zhengzhou University (2019-KY-357). The patients/participants provided their written informed consent to participate in this study.

## Author Contributions

SZ and JF contributed equally to this work, they were responsible for study design, data collection, and data analyzing. YS was involved in manuscript preparation and literature searching. HL took part in study design and manuscript revision. All the authors contributed to the article and approved the submitted version.

## Conflict of Interest

The authors declare that the research was conducted in the absence of any commercial or financial relationships that could be construed as a potential conflict of interest.

## Footnotes

1 https://www.gencodegenes.org/

2 https://www.ncbi.nlm.nih.gov/geo/

3 https://github.com/kevinblighe/PCAtools

4 http://www.microrna.org/

5 http://starbase.sysu.edu.cn/index.php

6 https://cm.jefferson.edu/

7 http://diana.imis.athena-innovation.gr/DianaTools/index.php

8 http://software.broadinstitute.org/gsea/msigdb/index.jsp

## Supplementary Material

FIGURE S1 Overall design and workflow of this study.

FIGURE S2 Soft threshold determination and the relationship between these two modules and clinical traits. **(A)** Determination of soft-thresholding power in Wgcna. **(B)** Scatter plot of module eigengenes in blue and green modules. **(C)** Module eigengene dendrogram and interactions among different gene coexpression modules.

FIGURE S3 Relative quantification of Linc00525 and Linc00996 expression by qRt-Pcr. The expression of Linc00525 **(A)** and Linc00996 **(B)** in human multiple myeloma cell lines (Rpmi-8226, Sko-007, Km3) as well as normal plasma cells. Data are presented as the mean ± standard deviation. The ns represents not significant, ^∗^ represents *P* < 0.05, ^∗∗^ represents *P* < 0.01, ^∗∗∗^ represents *P* < 0.001 and ^****^ represents *P* < 0.0001.

FIGURE S4 The risk score performance in the Tcga (testing) datasets. **(A)** Risk score of the 2 lncRnas in 787 Mm patients (top); overall survival status and duration (middle); heatmap of the 2 lncRnas expression in Mm patients (Bottom). **(B)** The forest plot showed the hazard ratios (Hr) with 95% confidence interval (95%Ci) according to the multivariate Cox regression results. **(C)** The overall survival of high-risk score group and low-risk score group. **(D)** The 3-year survival receiving operating characteristic curve (Roc) of according to 2 lncRna signature risk score (red).
