# Supplementary figures and images for: Integrated Analysis of a Risk Score System Predicting Prognosis and a ceRNA Network for Differentially Expressed lncRNAs in Multiple Myeloma
Source: Front Genet. 2020 Aug 27;11:934. doi: 10.3389/fgene.2020.00934 (PMC7481452; doi:10.3389/fgene.2020.00934)

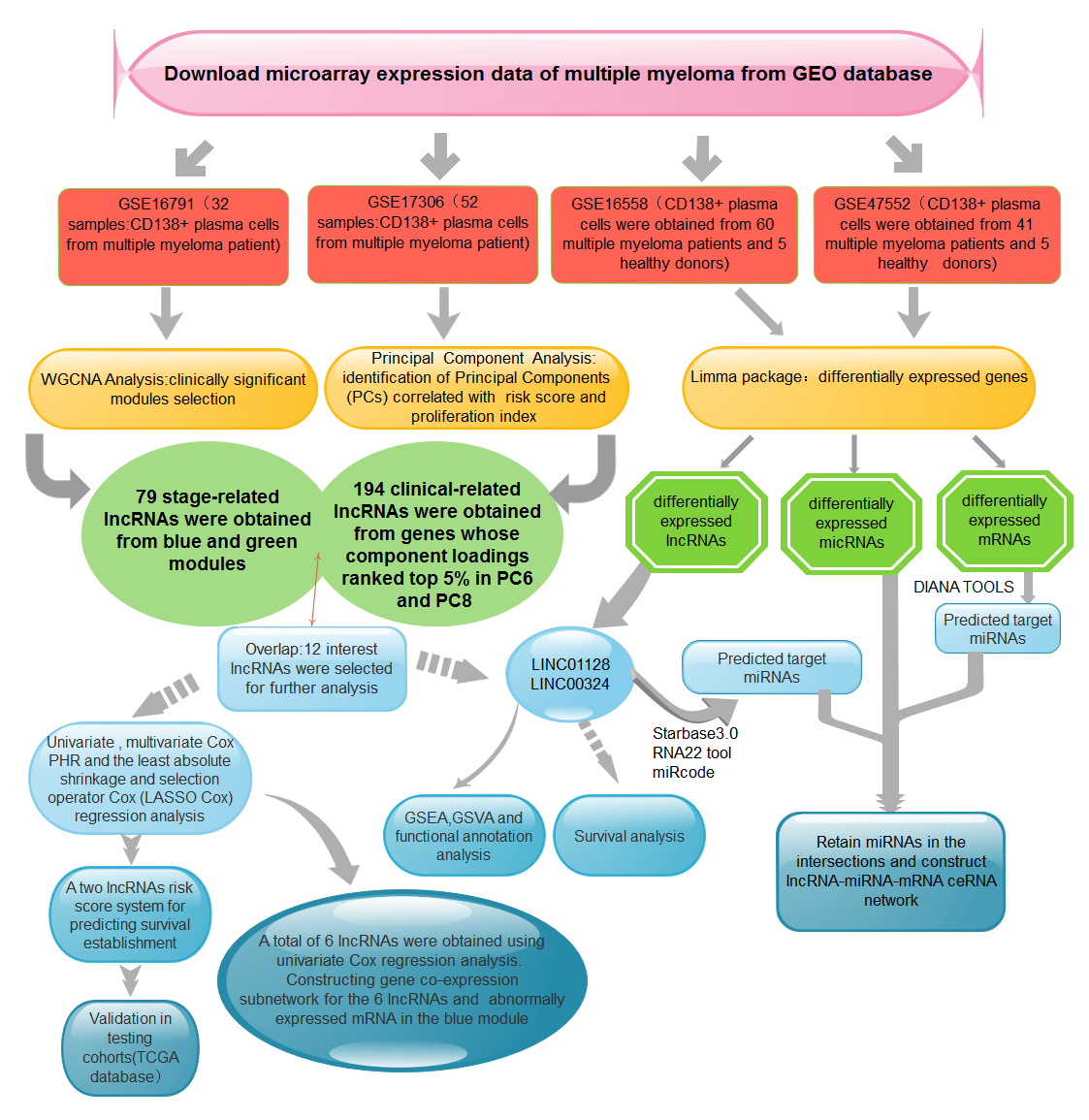

Supplement: FIGURE S1 — Overall design and workflow of this study. [file Image_1.TIFF]

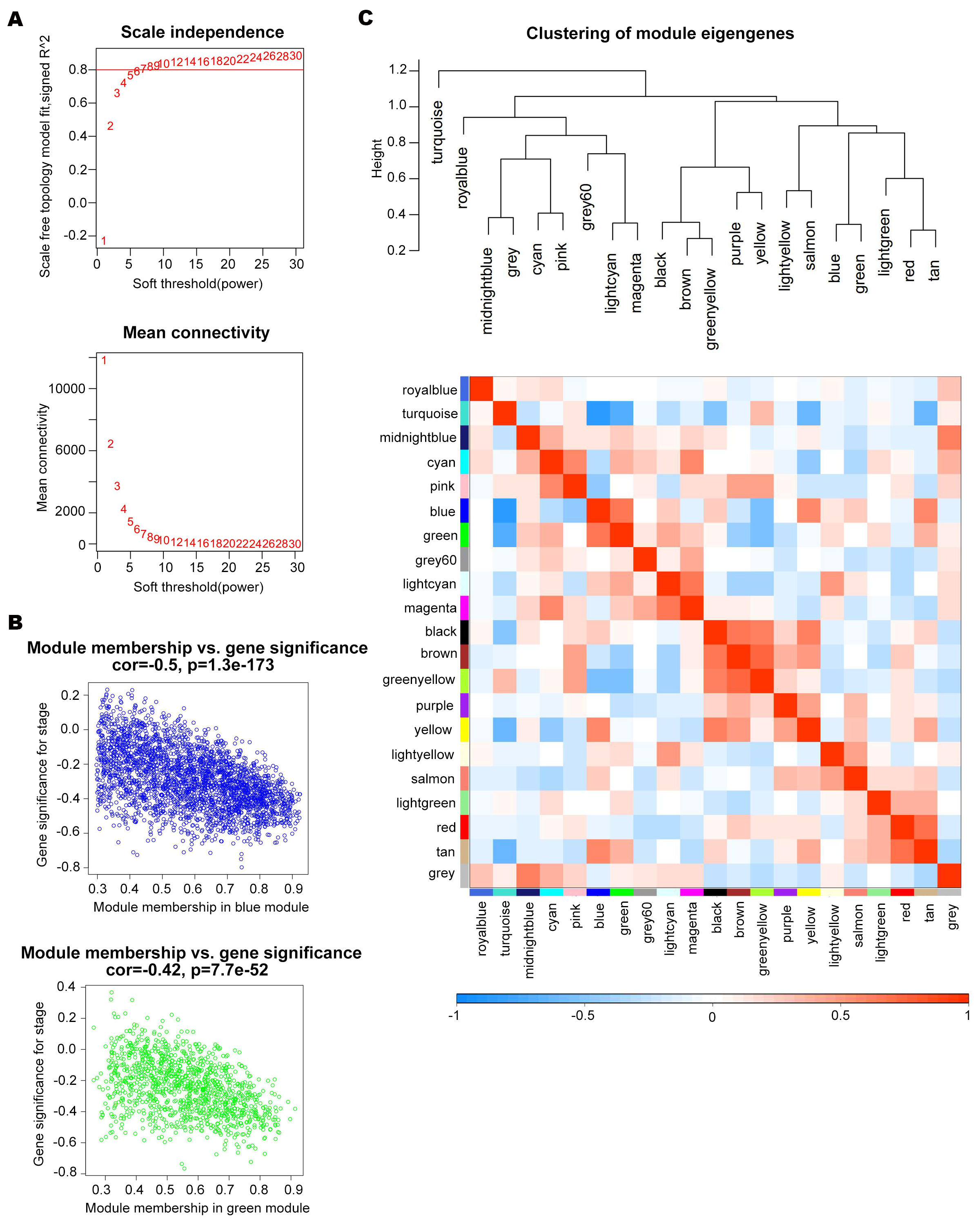

Supplement: FIGURE S2 — Soft threshold determination and the relationship between these two modules and clinical traits. (A) Determination of soft-thresholding power in Wgcna. (B) Scatter plot of module eigengenes in blue and green modules. (C) Module eigengene dendrogram and interactions among different gene coexpression modules. [file Image_2.TIF]

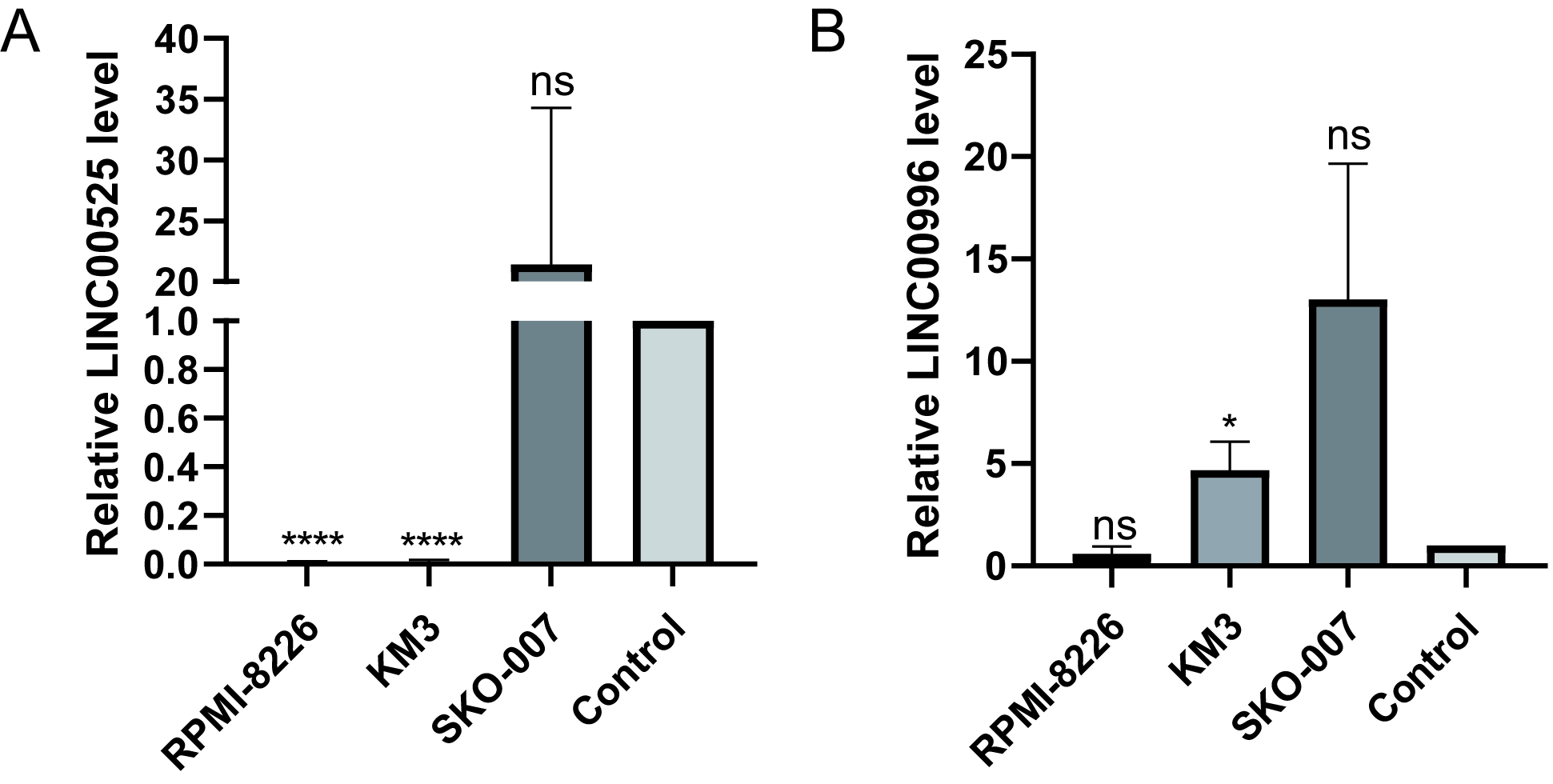

Supplement: FIGURE S3 — Relative quantification of Linc00525 and Linc00996 expression by qRt-Pcr. The expression of Linc00525 (A) and Linc00996 (B) in human multiple myeloma cell lines (Rpmi-8226, Sko-007, Km3) as well as normal plasma cells. Data are presented as the mean ± standard deviation. The ns represents not significant, ∗ represents P < 0.05, ∗∗ represents P < 0.01, ∗∗∗ represents P < 0.001 and **** represents P < 0.0001. [file Image_3.TIF]

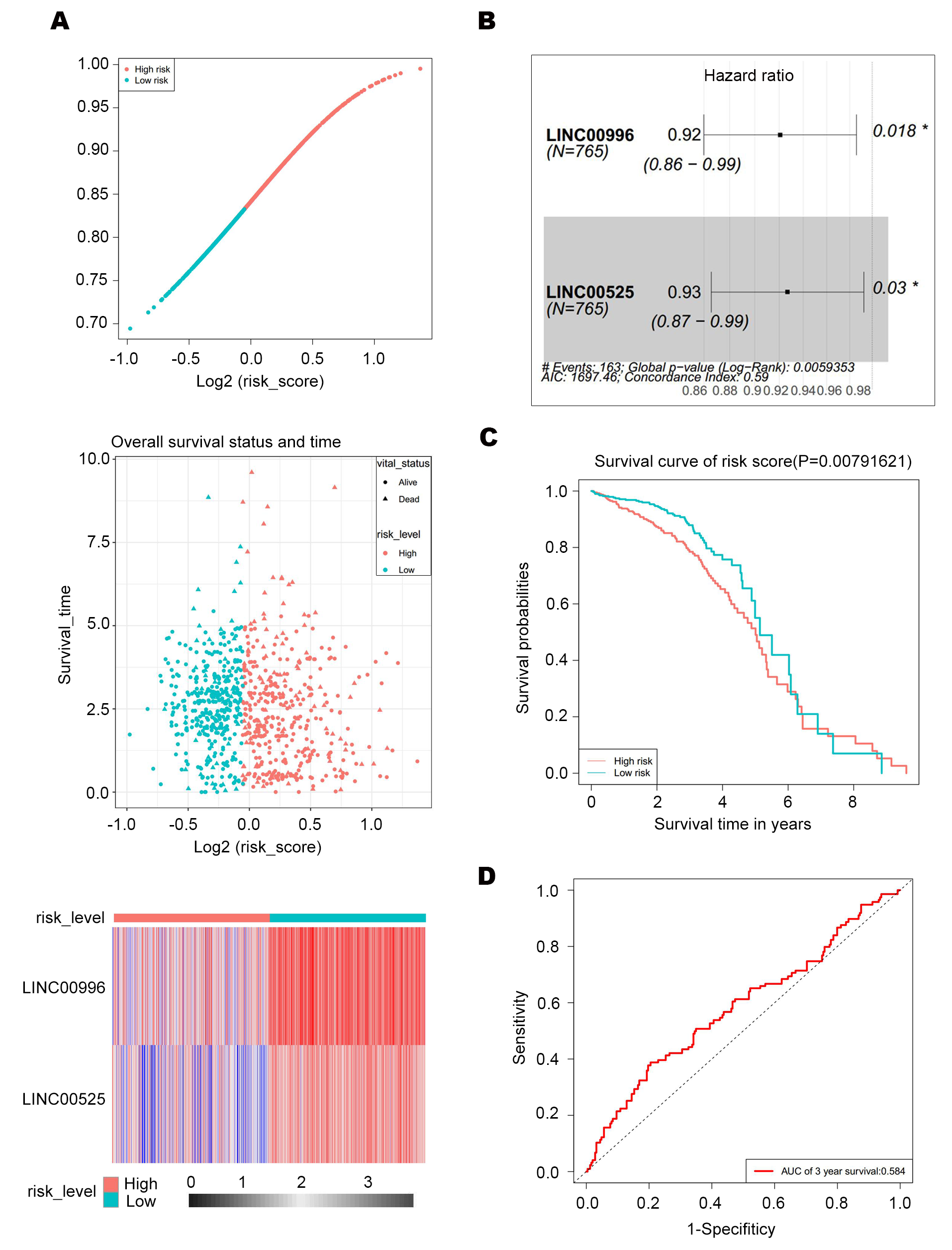

Supplement: FIGURE S4 — The risk score performance in the Tcga (testing) datasets. (A) Risk score of the 2 lncRnas in 787 Mm patients (top); overall survival status and duration (middle); heatmap of the 2 lncRnas expression in Mm patients (Bottom). (B) The forest plot showed the hazard ratios (Hr) with 95% confidence interval (95%Ci) according to the multivariate Cox regression results. (C) The overall survival of high-risk score group and low-risk score group. (D) The 3-year survival receiving operating characteristic curve (Roc) of according to 2 lncRna signature risk score (red). [file Image_4.TIF]
