# Supplementary material for: Integrated Analysis of a Risk Score System Predicting Prognosis and a ceRNA Network for Differentially Expressed lncRNAs in Multiple Myeloma
Source: Front Genet. 2020 Aug 27;11:934. doi: 10.3389/fgene.2020.00934 (PMC7481452; doi:10.3389/fgene.2020.00934)
Supplement: Supplementary file 5 [file Data_Sheet_1.zip › Supplementary Tables 1- 3.docx]

Supplementary Table S1: LASSO regression coefficients of two lncRNAs:

The remaining six lncRNAs were further analyzed using LASSO regression analysis, and cross-validation was adopted to select the penalty parameters. Two lncRNAs were identified by lambda.1se values. The LASSO regression coefficients of 2 lncRNAs were presented.

| lncRNA | Ensemble ID | LASSO Regression Coefficient |
| --- | --- | --- |
| LINC00525 | ENSG00000146666 | -0.071 |
| LINC00996 | ENSG00000242258 | -0.249 |

Supplementary Table S2: Univariate analysis and multivariate cox regression analysis for assessing the correlation of clinical factors and the lncRNA signature risk score with OS of MM patients in GSE57317 dataset.

| Variables | Univariate analysis | | Multivariate analysis | |
| --- | --- | --- | --- | --- |
|  | HR (95%CI) | P value | HR (95%CI) | P value |
| Molecular subtype | 1.161(1.077,1.252) | **<0.001** | 1.078(0.990,1.174) | 0.085 |
| Risk score | 1.868(1.324,2.635) | **<0.001** | 1.682(1.162,2.437) | **0.006** |

HR, hazard ratio; 95%; CI, 95% confidence interval.

Supplementary Table S3: Univariate analysis and multivariate cox regression analysis for assessing the correlation of clinical factors and the lncRNA signature risk score with OS of MM patients in TCGA dataset.

| Variables | Univariate analysis | | Multivariate analysis | |
| --- | --- | --- | --- | --- |
|  | HR (95%CI) | P value | HR (95%CI) | P value |
| Age | 1.020(1.005,1.036) | **0.009** | 1.011(0.996,1.026) | 0.148 |
| Gender | 0.791(0.565,1.108) | 0.172 | 0.782(0.559,1.095) | 0.153 |
| Stage | 1.749(1.425,2.148) | **<0.001** | 1.711(1.391,2.105) | **<0.001** |
| Risk score | 2.228(1.354,3.665) | **0.002** | 2.308(1.385,3.846) | **0.001** |

HR, hazard ratio; 95%; CI, 95% confidence interval.

Supplementary Table S4: Differentially expressed mRNAs in GSE16558 between CD138+ plasma cells obtained from 60 multiple myeloma patients and 5 healthy donors.

Supplementary Table S5: Potential DEmiRNAs that can target DEmRNAs co-expressed with LINC01128 and LINC00324 (Predicted by DIANA TOOLS)

Supplementary Table S6: LncRNA–miRNA–mRNA ceRNA network in MM.
